# Evaluating YouTube as a Source of Patient Education on the Role of the Hospitalist: A Cross-Sectional Study

**DOI:** 10.2196/ijmr.6393

**Published:** 2017-01-10

**Authors:** Tamer Hudali, Muralidhar Papireddy, Mukul Bhattarai, Alan Deckard, Susan Hingle

**Affiliations:** ^1^ Department of Internal Medicine Southern Illinois University School of Medicine Springfield, IL United States

**Keywords:** YouTube, hospitalist, patient education

## Abstract

**Background:**

Hospital medicine is a relatively new specialty field, dedicated to the delivery of comprehensive medical care to hospitalized patients. YouTube is one of the most frequently used websites, offering access to a gamut of videos from self-produced to professionally made.

**Objective:**

The aim of our study was to determine the adequacy of YouTube as an effective means to define and depict the role of hospitalists.

**Methods:**

YouTube was searched on November 17, 2014, using the following search words: “hospitalist,” “hospitalist definition,” “what is the role of a hospitalist,” “define hospitalist,” and “who is a hospitalist.” Videos found only in the first 10 pages of each search were included. Non-English, noneducational, and nonrelevant videos were excluded. A novel 7-point scoring tool was created by the authors based on the definition of a hospitalist adopted by the Society of Hospital Medicine. Three independent reviewers evaluated, scored, and classified the videos into high, intermediate, and low quality based on the average score.

**Results:**

A total of 102 videos out of 855 were identified as relevant and included in the analysis. Videos uploaded by academic institutions had the highest mean score. Only 6 videos were classified as high quality, 53 as intermediate quality, and 42 as low quality, with 82.4% (84/102) of the videos scoring an average of 4 or less.

**Conclusions:**

Most videos found in the search of a hospitalist definition are inadequate. Leading medical organizations and academic institutions should consider producing and uploading quality videos to YouTube to help patients and their families better understand the roles and definition of the hospitalist.

## Introduction

Hospitalist is a physician who specializes in delivering comprehensive medical care to hospitalized patients after receiving training in general internal medicine, general pediatrics, or family practice; however, he may also receive training in other medical disciplines [1,2]. Hospital medicine is a relatively new and evolving specialty field, dedicated to the delivery of comprehensive medical care to hospitalized patients. The term “hospitalist” was first described in literature by Wachter and Goldman in their article, The Emerging Role of “Hospitalists” in the American health care system [1]. They described this new specialty, its emergence, and their perspectives to the future. Now, hospital medicine is one of the fastest growing medical specialties. This rapid growth could be explained by the decreased length and cost of hospital stay under hospitalist care [3-7]. One study based on Medicare claims that its data showed an increase in the number of physicians identified as hospitalists from 5.9% to 19% between 1995 and 2006 [8]. The Society of Hospital Medicine defines a hospitalist as a physician who specializes in the practice of hospital medicine [2]. The role of the hospitalists has evolved over time, and it includes providing high-value care for hospitalized patients, conducting quality improvement projects, and adopting leadership roles, which have a positive impact on patients’ outcomes in terms of length and cost of hospital stay as well as readmission rates [1,9-11]. The perceived benefits have driven other specialties to adopt the hospitalist model [12].

As an emerging specialty, hospitalists face the difficulty of building a strong doctor-patient relationship. Building a rapport with patients is very important in clinical practice, as it enhances information gathering needed for diagnosis and is important for the shared-decision making process [13,14]. The hospital encounter is a short period to achieve this goal and patients lack insight into the role of a hospitalist. Furthermore, the communication barriers between the patient’s primary care physicians and the hospitalists can interrupt the ongoing doctor-patient relationship in the inpatient and outpatient settings [13]. This interruption in patient-provider relationship may result in lack of adequate communication and missing important information affecting patients’ outcome [15-18]. Unfortunately, few primary care and emergency department physicians inform patients about hospitalist coverage during their hospitalization [18,19]. This knowledge gap among patients can impede the therapeutic relationship and in turn negatively affect the patients’ outcome and liability risk [20-22].

The term “hospitalist” remains ambiguous to a majority of first-time hospitalized patients and their families. Because the Internet has become a popular source for health care information [23,24], we believe that people may search the Internet for the term “hospitalist” to clarify or obtain further information on physicians practicing this specialty. Similarly, hospitalized patients and their families are more likely to search the Internet for “hospitalists” in view of the current trend of shift from primary care physician to different inpatient provider in an era of easily accessible Internet on portable electronic devices. One study estimated that up to 70% of Internet users in the United States utilize the Internet for health-related searches [25,26]. Among the search engines, YouTube is the second largest after Google [27]. Over 6 billion hours of videos are watched each month on YouTube [28]. The video-based forum offers access to a gamut of self-produced and professionally made clips that have been uploaded and shared by individuals and groups. The accuracy and quality of contents of such videos vary widely. To our knowledge, there are no studies in the literature that highlight the overall usefulness of social media such as YouTube videos’ content in educating patients and families on hospital medicine and the role of the hospitalist. We sought to determine the adequacy and quality of using YouTube videos by the public as a way to define and depict the role of hospitalists.

## Methods

**Figure 1:**
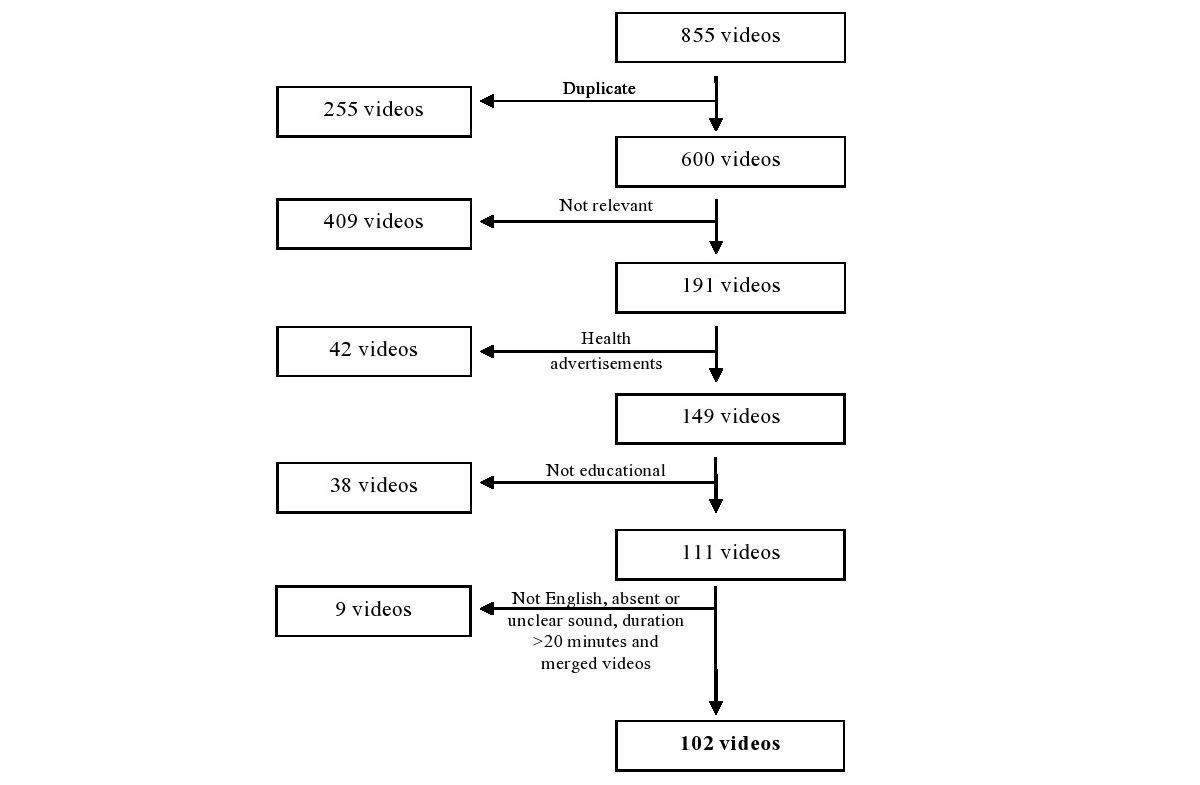
Flow diagram for selection of videos.

YouTube was searched on November 17, 2014, using the following search terms: “hospitalist,” “hospitalist definition,” “what is the role of a hospitalist,” “define hospitalist,” and “who is a hospitalist.” Videos found only in the first 10 pages of each search were included. A total of 855 videos were found. Non-English, noneducational, and nonrelevant videos were excluded, including the videos that lacked sound or were longer than 20 minutes. Duplicate videos were counted as one video. Using the inclusion criteria, we selected 102 videos for analysis. Selection process is depicted as a flowchart in Figure 1.

The selected videos were categorized according to uploader type (personal, academic institution, nonacademic institution, health advertisement, or news report); video category as per the YouTube classification (nonprofits & activism, people & blogs, science & technology, education, news & politics, and entertainment); and medical specialty (internal medicine, pediatrics, family medicine, obstetrics and gynecology, and others). We also collected the following information for each video: title, duration, number of views, likes and dislikes, upload date, and number of comments.

Next, a novel 7-point scoring tool was created by the authors based on the definition of a hospitalist adopted by the Society of Hospital Medicine (Table 1). Each measure describes an aspect or a characteristic role of hospitalists. The contents of the videos were evaluated based on the presence of the 7 measures depicted in the tool (Table 1). The information presented in the videos showed the appropriate implication depicted by the Society of Hospital Medicine’s definition for each measure to be eligible for a point. Three independent reviewers evaluated and scored the videos. The mean scores were used to classify the videos into high, intermediate, and low quality in defining hospitalists and their roles. A video was rated high if the average score was 5 or greater, intermediate for 3 or 4 points, and low quality for 2 or fewer.

Data were analyzed using SAS software version 9.4 (SAS institute Inc). We used the measure of central tendencies to express descriptive statistics. Data are presented as mean (SD). An intraclass correlation coefficient (ICC) was used to assess the reviewers’ performance.

**Table 1:** The 7-point scoring system to assess the quality and accuracy of the videos.

Quality and accuracy measure	Points
Defining the hospitalist as a physician who specializes in the practice of hospital medicine	1
Eligibility defined by residency training in general internal medicine, general pediatrics, or family medicine, but may also receive training in other medical disciplines	1
Prompt and complete attention to all patient care needs including diagnosis, treatment, and the performance of medical procedures (within their scope of practice)	1
Employing quality and process improvement techniques	1
Collaboration, communication, and coordination with all physicians and health care personnel caring for hospitalized patients	1
Safe transitioning of patient care within the hospital and from the hospital to the community, which may include oversight of care in postacute care facilities	1
Efficient use of hospital and health care resources	1

## Results

A total of 102 videos out of 855 were identified as relevant and included in the analysis. Videos were categorized by the source of uploader into nonacademic institution (private hospitals and hospitalist groups; 55.9%, 57/102), news reports (24.5%, 25/102), academic institutions (8.8%, 9/102), personal (5.9%, 6/102), health advertisements (3.9%, 4/102), and others (online medical dictionary explaining the word hospitalist; 1.0%, 1/102).

**Figure 2:**
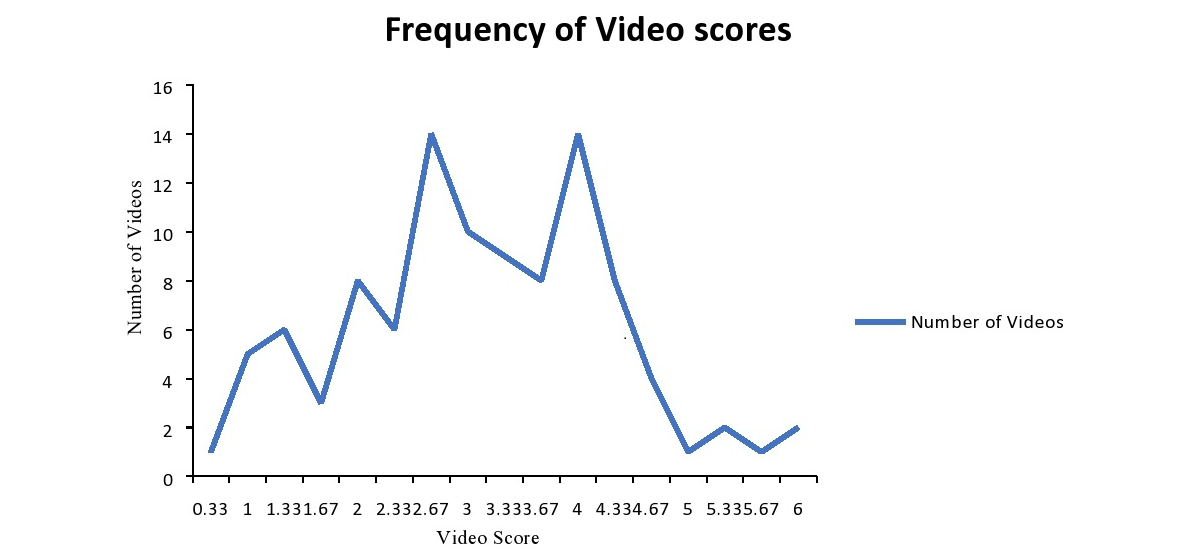
Frequency distribution of video scores.

After using our novel scoring tool, videos were classified into high, intermediate, and low quality. The average scores of the 3 reviewers (TH, MB, and MP) were 2.52, 3.46, and 3.36, respectively; the total average score for the 3 reviewers was 3.11 (SD 1.19). The interobserver agreement between the 3 reviewers showed an ICC of .809 (*P*<.001). Of the videos from all categories, 6 were classified as high quality, 53 as intermediate quality, and 42 as low quality, with 82.4% (84/102) of the videos scoring an average of 4 or less (Figure 2). The mean score of all videos was 3.11 (SD 1.19) with a minimum score of 0.33 and a maximum score of 6.0. The average number of views for the videos was 440.9 hits (SD 1401) with an average of 0.97 likes and 0.069 dislikes. The average duration of the videos was 3:17 minutes. Videos were uploaded between the years 2008 and 2014.

**Figure 3:**
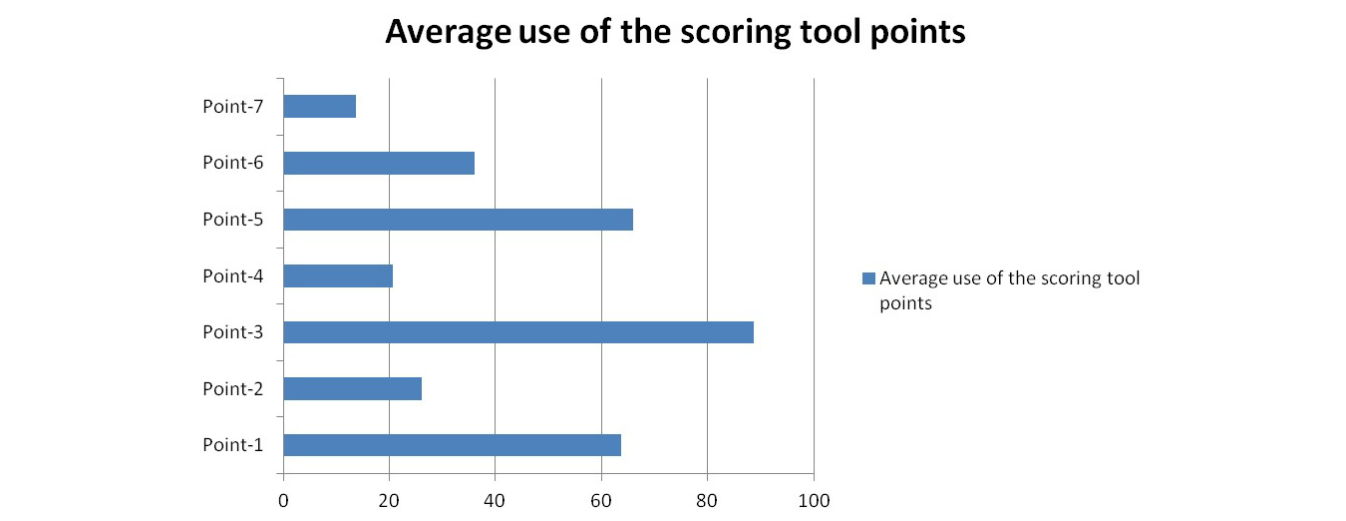
Average usage of the 7 scoring points.

Videos uploaded by academic institutions had the highest mean score of 3.37 (SD 0.73) and those uploaded by health advertisements and other media had the lowest. Table 2 shows the frequency and percentage of each category. Among the 7 scoring points of our scoring tool, point 3 addressing the hospitalist role in patient care including diagnosis, treatment, and the performance of medical procedures was seen most frequently on the videos. On the other hand, points 4 and 7 in our scoring tool were detected the least. These points addressed the hospitalists’ involvement in collaboration, communication, and coordination of care to hospitalized patients and the efficient utilization of health care resources, respectively. Figure 3 shows the average frequency of each point of the scoring tool.

**Figure 4:**
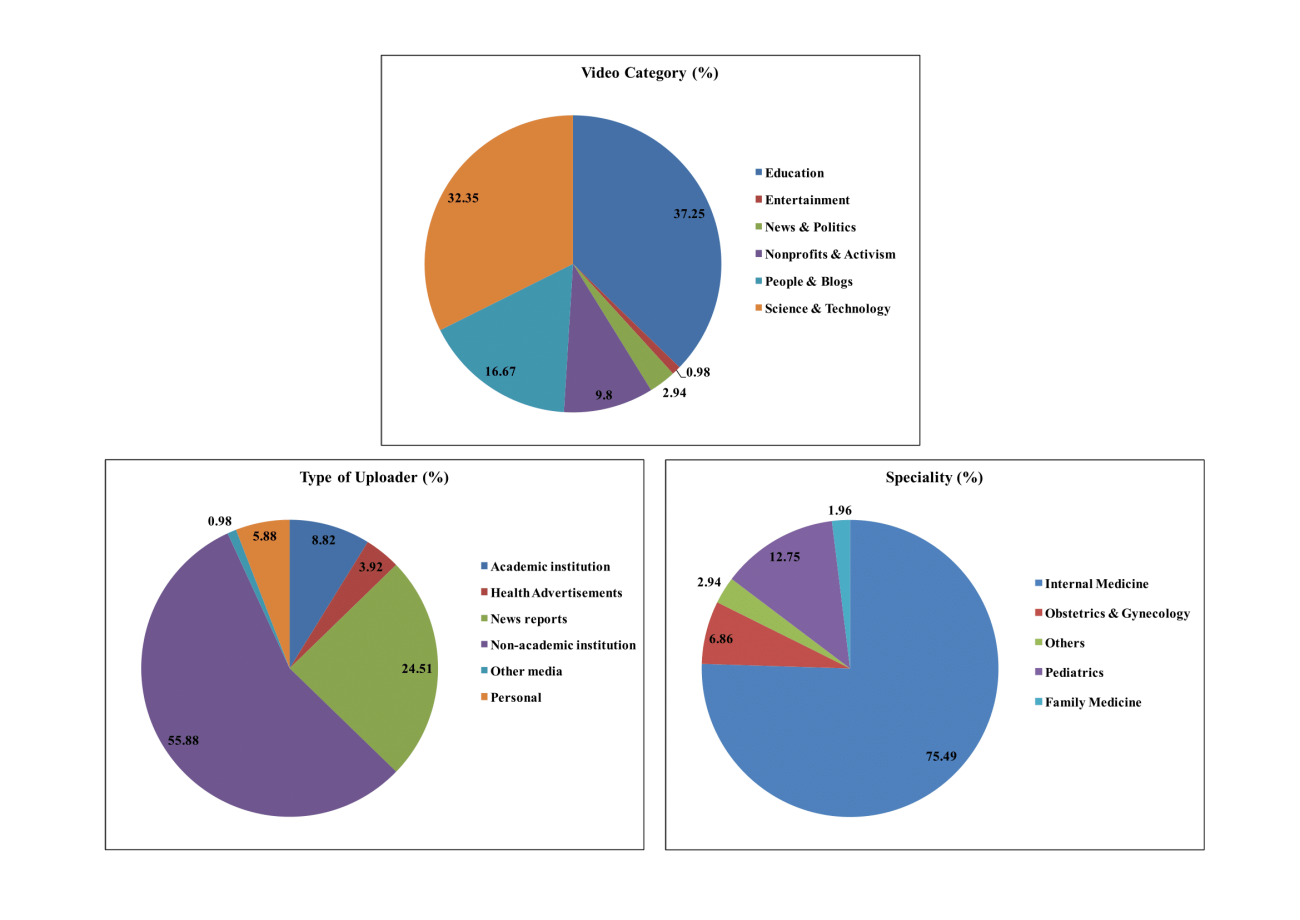
Distribution of the videos by video category, specialty, and source.

Videos were analyzed based on the YouTube category system. The videos came under the following 6 categories: Education (37.3%, 38/102), Science & Technology (32.3%, 33/102), People & Blogs (16.7%, 17/102), Nonprofits & Activism (9.8%, 10/102), News & Politics (2.9%, 3/102), and Entertainment (1.0%, 1/102). Figure 4 depicts the category distribution of the videos and the average scores by each category. The highest average score was for Nonprofits & Activism, and the lowest score was for Entertainment.

Next, we analyzed videos based on the specialty of hospitalist: internal medicine (75.5%, 77/102), pediatrics (12.7%, 13/102), Obstetrics and gynecology (6.9%, 7/102), family medicine (2.0%, 2/102) and others that included surgery and cardiology (2.9%, 3/102). Figure 4 demonstrates the specialty distribution and average scores by specialty.

**Table 2:** Source of the video.

Type of uploader	Frequency (N=102), n (%)
Nonacademic institutions	57 (55.9)
News reports	25 (24.5)
Academic institutions	9 (8.8)
Personal	6 (5.9)
Health advertisements	4 (3.9)
Other media	1 (1.0)

## Discussion

### Principal Findings

Health care information available in social media websites, such as YouTube, Facebook, MySpace, and Twitter, include accounts of personal illnesses, disease support groups, medical breakthroughs, updates in health and disease, journal articles, and clinical support tools for laypersons and health-related professionals [23,24]. Social media use has been increasing due to the advantages of its low cost, ease of publication, and interaction with a large community. Among the many types of social media and websites, YouTube remains the fastest growing. YouTube is considered the second most viewed website on the Internet [29]. Also, YouTube is the most visited and popular website for video-sharing in the United States for obtaining information. It is increasingly used as a platform to disseminate health care information and patient education. However, because there has been no quality check, the information that is available on YouTube can provide contradicting or misleading information to the layperson. Keelan et al [30] were among the first to analyze the quality of health care information in YouTube. Since then multiple studies have been published addressing the efficacy and quality of medical contents of the YouTube videos. To our knowledge, ours is the first study to assess the accuracy and usefulness of YouTube content in defining the role of hospitalists.

Hospital medicine is a relatively new specialty, leading patients and their families to potentially be puzzled the first time they encounter a hospitalist. The doctor-patient relationship forms the basis for optimal therapeutic and patient satisfaction outcomes [31,32]. With the increasing use of this specialty in hospitals, the patient experience is at jeopardy unless patients have made an informed decision to work with this new provider during the times of their utmost need. We believe that patients and their families do not fully understand the roles of this specialist, and they may search for further information on the Internet, particularly video-format sharing websites like YouTube. We conducted this study to evaluate the credibility of YouTube as a source of patient education on the role of the hospitalist.

YouTube has been used for providing health related information, but studies on YouTube contents have been published on only a few topics such as vaccination [30,33], tobacco use [34], breast-feeding [35], the influenza pandemic [36], basic life support [37], and acute myocardial infarction [38]. These studies show that health information found on YouTube can be misleading. A recent analysis was conducted to identify the measures used in studies assessing the quality of YouTube videos [39]. The study showed that multiple measures are used to evaluate the quality of video information including expert-driven, popularity-driven, or heuristic-driven measures. The authors finally concluded that caution should be applied when using YouTube for patient educational materials [39].

Our study shows that most uploaded videos were posted by media or as part of a news report and not related to any professional society, that is, mainly from the nonacademic institutions. Almost one half of the videos found on the primary search were deemed nonrelevant. Of the videos deemed relevant, none included all 7 points of our rating scale to completely define hospitalists and their roles. Most videos did not include the following points from our scoring tool: hospitalist involvement in quality improvement, efficient utilization of health care resources, and the qualifications required to become a hospitalist (Figure 3). A significant number of videos that described the hospitalist were uploaded solely to advertise hospitals or recruit hospitalists. However, videos uploaded by academic institutions received the highest mean score of 3.37 (SD 0.73), indicating a potential role for such institutions in using social media to provide an accurate definition of hospitalists and their roles. Kelly et al, [40] in their study of the content of YouTube in regard to nursing identity, showed similar results to our study. The authors concluded that professional bodies need to act to protect the nurses’ identity, representation, and job descriptions. Our study identifies the importance of social media websites and their potential usefulness for disseminating accurate information about the definition of hospitalist. During the process of hospital admission, the health care provider should communicate the definition and role of the hospitalist in providing and coordinating patient care to the patient and family. Video-sharing websites could serve as a powerful platform for dissemination of information on hospital medicine and the hospitalist.

### Study Limitations

This is a cross-sectional study. Content on YouTube changes constantly and more videos are uploaded daily. Furthermore, video optimization and analytics may also alter the search results. Also, this data is from a single video-broadcasting website on the Internet. The external validity of such data may be affected and may not project the scenario over the Internet as a whole.

### Conclusions

Most videos found in the search of a hospitalist definition are nonrelevant. Our study indicates the inadequacy of using YouTube as a tool in defining the role of hospitalists without some guidance in directing search engines toward the higher quality videos. Patients and families need to be cautious when using YouTube as a source for health-related information. Leading medical organizations and academic institutions should consider guiding the process of producing and uploading quality videos to YouTube to help patients and their families better understand the roles and definition of the hospitalist.

## References

[1] Wachter RM, Goldman L (1996). The emerging role of “hospitalists” in the American health care system. N Engl J Med.

[2] Hospitalmedicine.

[3] Rachoin J, Skaf J, Cerceo E, Fitzpatrick E, Milcarek B, Kupersmith E, Scheurer DB (2012). The impact of hospitalists on length of stay and costs: systematic review and meta-analysis. Am J Manag Care.

[4] Palmer HC, Armistead NS, Elnicki DM, Halperin AK, Ogershok PR, Manivannan S, Hobbs GR, Evans K (2001). The effect of a hospitalist service with nurse discharge planner on patient care in an academic teaching hospital. Am J Med.

[5] Kearns PJ, Wang CC, Morris WJ, Low DG, Deacon AS, Chan SY, Jensen WA (2001). Hospital care by hospital-based and clinic-based faculty: a prospective, controlled trial. Arch Intern Med.

[6] Meltzer D, Manning WG, Morrison J, Shah MN, Jin L, Guth T, Levinson W (2002). Effects of physician experience on costs and outcomes on an academic general medicine service: results of a trial of hospitalists. Ann Intern Med.

[7] Kuo Y, Goodwin JS (2011). Association of hospitalist care with medical utilization after discharge: evidence of cost shift from a cohort study. Ann Intern Med.

[8] Kuo Y, Sharma G, Freeman JL, Goodwin JS (2009). Growth in the care of older patients by hospitalists in the United States. N Engl J Med.

[9] Freed DH (2004). Hospitalists: Evolution, evidence, and eventualities. Health Care Manag.

[10] Kisuule F, Howell EE (2015). Hospitalists and their impact on quality, patient safety, and satisfaction. Obstet Gynecol Clin North Am.

[11] Wachter RM, Goldman L (2016). Zero to 50,000 - the 20th anniversary of the hospitalist. N Engl J Med.

[12] Nelson J, Wellikson L, Wachter R (2012). Specialty hospitalists: analyzing an emerging phenomenon. J Am Med Assoc.

[13] Barnett PB (2001). Rapport and the hospitalist. Am J Med.

[14] Hall K, Gibbie T, Lubman DI (2012). Motivational interviewing techniques - facilitating behaviour change in the general practice setting. Aust Fam Physician.

[15] Pham HH, Grossman JM, Cohen G, Bodenheimer T (2008). Hospitalists and care transitions: the divorce of inpatient and outpatient care. Health Aff (Millwood).

[16] Roy CL, Poon EG, Karson AS, Ladak-Merchant Z, Johnson RE, Maviglia SM, Gandhi TK (2005). Patient safety concerns arising from test results that return after hospital discharge. Ann Intern Med.

[17] Hinami K, Farnan JM, Meltzer DO, Arora VM (2009). Understanding communication during hospitalist service changes: a mixed methods study. J Hosp Med.

[18] Kripalani S, LeFevre F, Phillips CO, Williams MV, Basaviah P, Baker DW (2007). Deficits in communication and information transfer between hospital-based and primary care physicians: implications for patient safety and continuity of care. J Am Med Assoc.

[19] Hesselink G, Schoonhoven L, Barach P, Spijker A, Gademan P, Kalkman C, Liefers J, Vernooij-Dassen M, Wollersheim H (2012). Improving patient handovers from hospital to primary care: a systematic review. Ann Intern Med.

[20] Forster AJ, Clark HD, Menard A, Dupuis N, Chernish R, Chandok N, Khan A, van Walraven C (2004). Adverse events among medical patients after discharge from hospital. CMAJ.

[21] Moore C, Wisnivesky J, Williams S, McGinn T (2003). Medical errors related to discontinuity of care from an inpatient to an outpatient setting. J Gen Intern Med.

[22] Coleman EA, Min S, Chomiak A, Kramer AM (2004). Posthospital care transitions: patterns, complications, and risk identification. Health Serv Res.

[23] Vance K, Howe W, Dellavalle RP (2009). Social internet sites as a source of public health information. Dermatol Clin.

[24] McNab C (2009). What social media offers to health professionals and citizens. Bull World Health Organ.

[25] Sadasivam RS, Kinney RL, Lemon SC, Shimada SL, Allison JJ, Houston TK (2013). Internet health information seeking is a team sport: analysis of the Pew Internet Survey. Int J Med Inform.

[26] Dickerson S, Reinhart AM, Feeley TH, Bidani R, Rich E, Garg VK, Hershey CO (2004). Patient Internet use for health information at three urban primary care clinics. J Am Med Inform Assoc.

[27] Hubspot.

[28] Youtube.

[29] Alexa.

[30] Keelan J, Pavri-Garcia V, Tomlinson G, Wilson K (2007). YouTube as a source of information on immunization: a content analysis. J Am Med Assoc.

[31] Stewart MA (1995). Effective physician-patient communication and health outcomes: a review. Can Med Assoc J.

[32] Kaplan SH, Greenfield S, Ware JE (1989). Assessing the effects of physician-patient interactions on the outcomes of chronic disease. Med Care.

[33] Ache KA, Wallace LS (2008). Human papillomavirus vaccination coverage on YouTube. Am J Prev Med.

[34] Freeman B, Chapman S (2007). Is “YouTube” telling or selling you something? Tobacco content on the YouTube video-sharing website. Tob Control.

[35] Eglash A (2009). Website review: www.Youtube.com. Breastfeed Med.

[36] Pandey A, Patni N, Singh M, Sood A, Singh G (2010). YouTube as a source of information on the H1N1 influenza pandemic. Am J Prev Med.

[37] Yaylaci S, Serinken M, Eken C, Karcioglu O, Yilmaz A, Elicabuk H, Dal O (2014). Are YouTube videos accurate and reliable on basic life support and cardiopulmonary resuscitation?. Emerg Med Australas.

[38] Pant S, Deshmukh A, Murugiah K, Kumar G, Sachdeva R, Mehta JL (2012). Assessing the credibility of the “YouTube approach” to health information on acute myocardial infarction. Clin Cardiol.

[39] Gabarron E, Fernandez-Luque L, Armayones M, Lau AY (2013). Identifying measures used for assessing quality of YouTube videos with patient health information: a review of current literature. Interact J Med Res.

[40] Kelly J, Fealy GM, Watson R (2012). The image of you: constructing nursing identities in YouTube. J Adv Nurs.

